# A Novel Hybrid Mental Spelling Application Based on Eye Tracking and SSVEP-Based BCI

**DOI:** 10.3390/brainsci7040035

**Published:** 2017-04-05

**Authors:** Piotr Stawicki, Felix Gembler, Aya Rezeika, Ivan Volosyak

**Affiliations:** Faculty of Technology and Bionics, Rhine-Waal University of Applied Sciences, 47533 Kleve, Germany; piotr.stawicki@hochschule-rhein-waal.de (P.S.); felix.gembler@hochschule-rhein-waal.de (F.G.); aya.rezeika@hsrw.org (A.R.)

**Keywords:** Brain-Computer Interface (BCI), Electroencephalogram (EEG), Steady State Visual Evoked Potential (SSVEP), Eye Tracking, Hybrid BCI

## Abstract

Steady state visual evoked potentials (SSVEPs)-based Brain-Computer interfaces (BCIs), as well as eyetracking devices, provide a pathway for re-establishing communication for people with severe disabilities. We fused these control techniques into a novel eyetracking/SSVEP hybrid system, which utilizes eye tracking for initial rough selection and the SSVEP technology for fine target activation. Based on our previous studies, only four stimuli were used for the SSVEP aspect, granting sufficient control for most BCI users. As Eye tracking data is not used for activation of letters, false positives due to inappropriate dwell times are avoided. This novel approach combines the high speed of eye tracking systems and the high classification accuracies of low target SSVEP-based BCIs, leading to an optimal combination of both methods. We evaluated accuracy and speed of the proposed hybrid system with a 30-target spelling application implementing all three control approaches (pure eye tracking, SSVEP and the hybrid system) with 32 participants. Although the highest information transfer rates (ITRs) were achieved with pure eye tracking, a considerable amount of subjects was not able to gain sufficient control over the stand-alone eye-tracking device or the pure SSVEP system (78.13% and 75% of the participants reached reliable control, respectively). In this respect, the proposed hybrid was most universal (over 90% of users achieved reliable control), and outperformed the pure SSVEP system in terms of speed and user friendliness. The presented hybrid system might offer communication to a wider range of users in comparison to the standard techniques.

## 1. Introduction

Brain-computer interfaces (BCIs) can provide a communication channel without the involvement of muscular activity [1,2]. Through the detection of specific brain patterns, in the noninvasively acquired electroencephalogram (EEG) data, users are enabled to perform direct commands in real time. BCIs have therefore the potential to be utilized as assistive technology for people with restricted motor abilities.

In this article we present a communication system that is based on the Steady-State Visually Evoked Potential (SSVEP) BCI paradigm [3,4,5]. SSVEP-based BCIs can be categorized as reactive BCI paradigm as it is based on the response to an external stimuli. Potentials are evoked at a certain frequency if the gaze is fixated on a flickering target at the same frequency.

Though SSVEP-based BCIs have been proven to be usable by most, if not all healthy users [6,7], there is an ongoing debate and concern regarding its dependency on eye gaze, which excludes patients with lack of oculomotor control from using such systems. Although some researchers address this issue showing that SSVEPs can also be controlled by shifting attention rather than gaze and striving towards so-called independent SSVEP-based BCI [8,9,10], the majority of studies—to some extend—allow gaze shift. Such systems then compete with other healthcare applications based on gaze direction such as eye trackers.

Eye trackers are devices which calculate gaze coordinates on the screen according to measured eye positions. So called video occulography eye trackers use a video camera which can be positioned in front of a subject or be head mounted (e.g., on eyeglasses frame), to track the position of the eyes [11,12]. Eye trackers are considered as robust and are already an established technology as communication systems for disabled persons; commercial systems such as the Tobii eye tracking devices (Tobii AB, Danderyd, Stockholm, Sweden) have become a valuable tool in augmentative communication [13,14,15].

The general consensus seems to be that eye tracking devices generally outperform SSVEP BCIs as they are faster and the required setup is much simpler. However, some studies suggest that the performance gap between the two technologies might be smaller than expected. Kishore et al. compared the two methods in a head-mounted display (HMD) hardware as a means of controlling gestures of a humanoid robot [16]. They found that both methods are appropriate for usage in immersive settings. All ten SSVEP participants triggered at least one gesture during the test. However, results for the Eye Tracker were surprisingly poor. Two out of ten eye tracker participants did not succeed in triggering gestures with the robot. They stated though, that there were technological differences in their setup compared to existing literature. Kos’myna and Tarpin-Bernard tested eye tracking in combination with different BCI paradigms in a gaming setup. Although they observed that the combination of eye tracking and SSVEP was slightly slower, it was more accurate than the pure eye tracker [17]. They concluded that the combination of the eye tracker and SSVEP was a well-rounded and natural combination.

One major obstacle with the eye tracking technology is, the so called, Midas touch problem (see e.g., [18]). Usually the activation of a selected target object is based on dwell times; the user has to focus on a target object for an extended period. But the system cannot differentiate intentional from unintentional fixation, which can easily lead to false classifications. One way to solve this problem is to use a BCI for target activation (see e.g., [19]).

For SSVEP-based BCIs another problem can occur. If consecutive commands are performed in a row (e.g., in spelling) the user needs to shifts her/his gaze between targets. EEG data collected during this gaze shifting phase is not relevant for the identification of any stimuli. That is why many researchers include automatic pauses after classifications in their applications to give the user a fixed amount of time to find the next target. However, the time needed to find the next target, depends on their arrangement, the familiarity with the application, as well as user factors, hence, the length of the pause provided by the system might not be optimal and slow the system down. A more user specific pause for gaze shifting could be determined with eye tracking.

If gaze control is not restricted, it is convenient to combine BCIs and eye tracking devices. Several hybrid systems combining eye trackers with BCI approaches have been developed with applications for controlling robotic limb prosthetics [20], quadrocopters [21], games [17] and communication tools [19,22,23].

Several hybrid systems utilize a BCI as a supportive technology for eye tracking. In such technologies, usually the eye tracking device is used for selection and the BCI for the activation of an object, circumventing the Midas touch problem described above. E.g., Vilmek and Zander proposed a spelling system using eye tracking for target selection and an imaginary movement based BCI for simulating a mouse click [19]. They stated that their hybrid system was somewhat slower but more reliable in comparison to standard dwell time based eye tracking interfaces. However, no direct comparisons between the BCI and the eye tracking systems were made.

McCullagh et al. used an EyeTribe tracker (The Eye Tribe ApS, Copenhagen, Denmark) for gaze coordinates and EEG-data recorded with an Emotiv EPOC (Emotiv, San Francisco, CA, USA) for selection [24]. Their hybrid approach maintained information transfer rate (ITR) while accuracy and efficiency were increased. They also stated that the ITR of both, the tested eye tracking device and the hybrid were higher in comparison to a previous SSVEP-only system.

Another approach is to use the eye tracker component as a complementary technology to the BCI. Lim et al. used the information of eye gaze direction detected by a low cost web-cam to prevent typing errors in an SSVEP-based BCI spelling application [23]. In online experiments with 10 participants, almost 40% of typos were prevented which shows that their system could reduce typing errors significantly.

The aforementioned methods have a clear distribution of tasks whereas the hybrid proposed in this article utilizes a more balanced allocation of tasks between the eye tracker and the BCI.

The here presented novel system allows hand-free control over a 30 target spelling interface using eye tracking for initial rough selection and the SSVEP technology for fine selection and activation. As we found during previous research, SSVEP systems with four or less targets allow high classification rates and offer control to a wide range of users [7]. Therefore, in this hybrid system we implemented only four simultaneously flickering stimuli. The letters are arranged in a 6 × 5 target matrix. If the user focuses on a specific letter, the area of the desired target is determined via eye tracking and a block of four letters starts flickering. As each of these four letters has a specific individual stimulation frequency, the system is able to classify a command. Gaze coordinates are tracked simultaneously in the background, allowing the user to switch to another block of letters if the initial area is false.

This method has several advantages:

- Eye tracking data is not used for activation of letters, the Midas touch problem is circumvented.
- Dynamic gaze shifting phases, ensuring that EEG data are only considered if the target object is fixated.
- Only four SSVEP stimuli need to be distinguished resulting in high classification accuracy.
- Little precision is expected from the eye tracking device, allowing a low cost hardware solution.

The presented article evaluates the feasibility of the proposed system and compares its performance to a pure SSVEP as well as a pure eye tracking system. In this respect a 30-target user interface was implemented for each of the three approaches.

## 2. Experimental Section

### 2.1. Participants

In total 32 able-bodied volunteer participants (six female) with mean (SD) age of 25.16 (7.71) years, ranging from 19 to 63 were recruited from the Rhine-Waal University of Applied Sciences (Kleve, Germany). Participants had normal or corrected-to-normal vision and had little to no previous experience with BCI systems. They gave written informed consent in accordance with the Declaration of Helsinki before taking part in the experiment. This research was approved by the ethical committee of the medical faculty of the University Duisburg-Essen. Information needed for the analysis of the test was stored pseudonymously. The entire session lasted on average approximately 50 min. Participants had the opportunity to withdraw at any time.

The EEG recordings were conducted in a quiet laboratory setting; luminance was kept low. Participants did not receive any financial reward.

### 2.2. Hardware

Participants were seated in front of a LCD screen (BenQ XL2420T, Taipei, Taiwan, resolution: 1920×1080 pixels, vertical refresh rate: 120 Hz) at a distance of about 60 cm. The used computer system operated on Microsoft Windows 7 Enterprise (Redmind, WA, USA) running on an Intel processor (Intel Core i7, Santa Clara, CA, USA. 3.40 GHz). An electroencephalogram (EEG) amplifier, g.USBamp (Guger Technologies, Graz, Austria) with standard Ag/AgCl electrodes were utilized. Eight signal electrodes were located over the visual cortex ($P_{Z},PO_{3},PO_{4},O_{1},O_{2},O_{Z},O_{9}$ and $O_{10}$ in accordance with the international system of EEG electrode placement). The ground electrode was placed over $AF_{Z}$, the reference electrode over $C_{Z}$. Standard abrasive electrolytic electrode gel was applied between the electrodes and the scalp in order to bring impedances below 5 kΩ. An analogue bandpass filter between 2 and 30 Hz and a notch filter of around 50 Hz were applied in the g.USBamp amplifier.

For the eye tracking aspect, we used the low cost EyeTribe tracker with the provided software development kit. The EyeTribe is a video-based tracker, which uses binocular gaze data and high resolution infrared LED illumination [25]. The data rate of the EyeTribe was set to 30 Hz. The software development kit provides a calibration interface which ensures a correct position of the device and identifies unique eye characteristics needed to enhance the accuracy of the tracker. The EyeTribe tracker was mounted on a tripod and was placed in front of the monitor, facing the user. It was connected to the computer via the universal serial bus (USB 3.0 port).

### 2.3. Signal Processing

For SSVEP signal classification, the minimum energy combination method (MEC) as proposed by Friman et al. in [26] was utilized. The MEC creates a set of channels (a weighted combination of the electrode signals) which minimize the nuisance signals. Considering $N_{t}$ samples of EEG data, recorded for each of $N_{y}$ signal electrodes, the SSVEP response for a flickering stimuli of *f* Hz, measured with the *i*-th electrode, can be described as function of the frequency *f* and its harmonics *k*, with corresponding amplitudes $a_{i,k}$ and $b_{i,k}$:

(1) $$y_{i}\left(t\right)\;=\;\sum_{k=1}^{N_{h}}a_{i,k}\sin\left(2\pi kft\right)+b_{i,k}\cos\left(2\pi kft\right)+E_{i,t}$$

The term $E_{i,t}$ represents the noise component of the electrode *i*, the various artifacts that cannot attribute to the SSVEP response. For a time segment length of $T_{s}$, acquired with a sampling frequency of $F_{E}$ Hz, the model can be described in a vector form as $y_{i}=X\tau_{i}+E_{i}$ where $y_{i}={\left[y_{i}\left(1\right),\ldots ,y_{i}\left(N_{t}\right)\right]}^{T}$ and *X* describes the $N_{t}\times 2N_{h}$ SSVEP model matrix containing the sine and cosine components. Further, the vector $\tau_{i}$ contains the corresponding amplitudes $a_{i,k}$ and $b_{i,k}$.

To cancel out the nuisance and noise, $N_{s}$ channel vectors $s_{i},\;i=1,\ldots ,N_{s}$ of length $N_{t}$ are defined as a linear combination of the electrode signals; the $N_{t}\times N_{s}$ matrix $S=[s_{1},\ldots ,s_{N_{s}}]$ can be written as S=XW, where the $N_{t}\times N_{s}$ matrix *W* contains the corresponding weights.

The noise and nuisance signals can be estimated by removing the SSVEP components from the signal. In this respect, the signal *Y* is projected on the orthogonal complement of the SSVEP model matrix,

(2) $$\tilde{Y}=Y-X{\left(X^{T}X\right)}^{-1}X^{T}Y.$$

As $B\approx \tilde{Y}$, an optimal weight combination for the electrode signals can then be found by calculating the eigenvectors of the symmetric matrix ${\tilde{Y}}^{T}\tilde{Y}$ (please refer to [27] for more details).

The weight matrix can be set to $W=\left[\frac{v_{1}}{\sqrt{\lambda_{1}}}\ldots \frac{v_{N_{s}}}{\sqrt{\lambda_{N_{s}}}}\right]$, where $\lambda_{1}\leq \lambda_{2}\leq \ldots \lambda_{N_{s}}$ are the calculated eigenvalues with corresponding eigenvectors $v_{i}$.

To discard up to 90% of the nuisance signal the total number of channels is selected by finding the smallest value for $N_{s}$ that satisfies the equation:

(3) $$\frac{\sum_{i=1}^{N_{s}}\lambda_{i}}{\sum_{j=1}^{N_{y}}\lambda_{j}}>0.1.$$

To detect the SSVEP response for a specific frequency, the power of that frequency and its harmonics $N_{h}$ is estimated by

(4) $$\hat{P}\;=\;\frac{1}{N_{s}N_{h}}\sum_{l=1}^{N_{s}}\sum_{k=1}^{N_{h}}{∥X_{k}^{T}s_{l}∥}^{2}.$$

The SSVEP power estimations of all $N_{f}$ considered frequencies are then normalized,

$$p_{i}\;=\;\frac{\hat{P_{i}}}{\sum_{j=1}^{N_{f}}{\hat{P}}_{j}}.$$

Finally, in order to highlight the largest values, a Softmax function was applied as described in [28],

(5) $$p_{i}^{\prime }\;=\;\frac{e^{\alpha p_{i}}}{\sum_{j=1}^{j=N_{f}}e^{\alpha p_{j}}},$$

where α was set to 0.25.

### 2.4. Software

**Figure 1:**
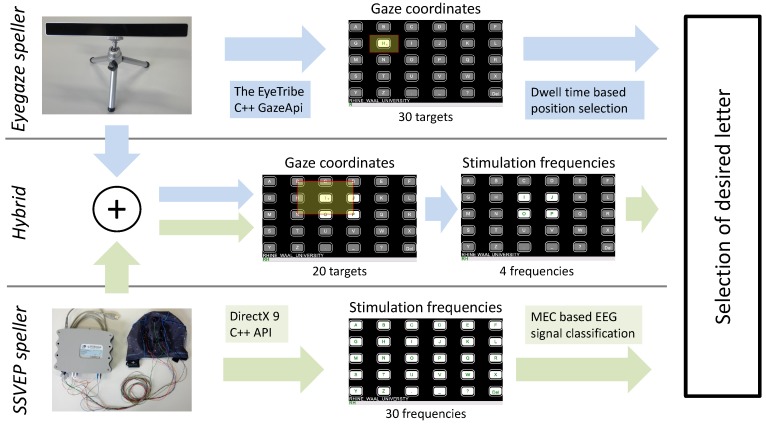
System overview of the three tested applications showing hardware, signal processing options, user interfaces and the system output.

The EEG signal classification and processing, as well as the graphical user interface, were implemented as Microsoft Visual Studio C++ project (Version 2010, Redmond, WA, USA). For eye tracking, source files from the C++ GazeApi library provided by the Eyetribe C++ software development kit (SDK) were included manually into the project. Three different spelling applications were tested in the experiment: the *SSVEP speller*, solely based on the SSVEP paradigm, the *Eyegaze speller*, solely based on eye tracking, and the *Hybrid*, a combination of both control technologies. Figure 1 provides a system overview of the tested applications. In each application, thirty boxes containing the alphabet plus additional special characters were presented to the user. Command classifications were followed by an audio feedback voicing the selected command. Table 1 summarizes the main characteristics of each interface. A detailed description of each speller is provided in the following.

*SSVEP speller*: For the *SSVEP speller*, as well as the *Hybrid*, the MEC was utilized as described above. To avoid overlapping of frequencies, $N_{h}=2$ harmonic frequencies were considered. For the *SSVEP speller*, power estimations for $N_{f}=30$ frequencies were calculated. Each block consisted of 13 samples (101.5625 ms with the sampling rate of 128 Hz). For the on-line classification, we used block-wise increasing classification time windows instead of sliding windows, as we learned that some users benefit from larger time segments (see e.g., [7]). If a particular stimulation frequency had the highest probability, exceeded a certain predefined threshold and the classification time window exceeded a certain minimum threshold, the corresponding command was classified. As more frequencies needed to be distinguished, the minimum classification time windows was set to 20 blocks (approximately 2 s), in order to avoid false classifications. After each performed classification the flickering stopped for approximately 914 ms (9 blocks) and no EEG data were collected, so that during this gaze shifting period, the user had time to shift her/his gaze to another target.

In the *SSVEP speller*, each box represented a stimulation frequency; the box size varied (between 130 × 90 and 170 × 120 pixels) in relation to the SSVEP power estimations during the experiment as described in [29]. Each box was outlined by a frame determining the maximum box size which was reached immediately prior to the classification.

To implement the 30 stimulation frequencies, a frame-based stimulus approximation was used (see e.g., [30,31]). In the frame-based stimulus approximation method, a varying number of frames is used in each cycle. The stimulus signal at frequency *f* is generated by

stim(f,i)=square[2πf(i/RefreshRate)],

where square(2πft) generates a square wave with frequency *f* and *i* is the frame index. E.g., the black/white reversing interval for the approximated frequency 17 Hz includes 17 cycles of varying length (three or four frames). For example, by using the formula above, the one-second stimulus sequence of 17 Hz can be generated: (*4 4* 3 4 3 4 3 4 3 4 3 4 3 4 3 4 3 *4 4* 3 4 3 4 3 4 3 4 3 4 3 4 3 4 3).

**Figure 2:**
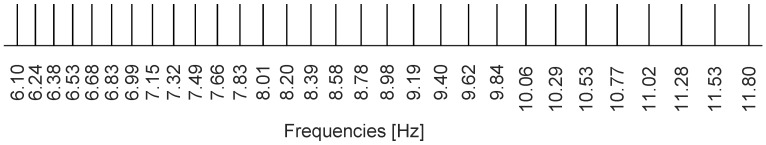
A logarithmically spaced set of frequencies used for the steady-state visual evoked potentials based application (*SSVEP speller*). Frequencies were implemented on the basis of the frequency approximation method.

For the online spelling task with the *SSVEP speller*, approximated frequencies between 6.1 and 11.8 Hz (resolution < 0.2 Hz) were used. This interval was applied in previous studies as well, because it avoids overlapping in the 2-nd harmonics frequencies while still allowing a sufficient difference in-between [32]. As indicated in [33], an equidistant stimuli set is not optimal, hence we selected 30 logarithmically spaced frequencies as displayed in Figure 2.

*Eyegaze speller*: This application used the eye movements as input modality. Each box could be selected by looking at it. The selected box was highlighted white, while the 29 remaining boxes were grey.

The EyeTribe tracker calculated gaze coordinates e=(x,y) with respect to the monitor at which the participants were looking at. In order to detect the gaze box a user was focusing on, exponentially weighted moving averages (EWMAs) were utilized. In the EWMA, a weighted average between the current value and the average of the last observation is averaged. For a series of sample coordinate vectors $e_{t}$ the EWMA was calculated recursively:

(6) $$\begin{array}{c} {\hat{e}}_{1}=e_{1},\;{\hat{e}}_{t}=\left(1-\alpha \right){\hat{e}}_{t-1}+\alpha e_{t},\;\mathrm{for}t>1. \end{array}$$

We used α=0.0625 to put more weight on the past value. After a time window of 0.33 s (10 sample coordinates), the box with the minimum distance to the average gaze position $\hat{e}={\hat{e}}_{10}$ was highlighted white, however, in order to spell the letter contained in the box, it needed to be classified three times in a row. Hence the minimum time to select a character was 1 s. After a letter selection, letter classification was suppressed for the duration of 2 s to avoid false classifications.

**Figure 3:**
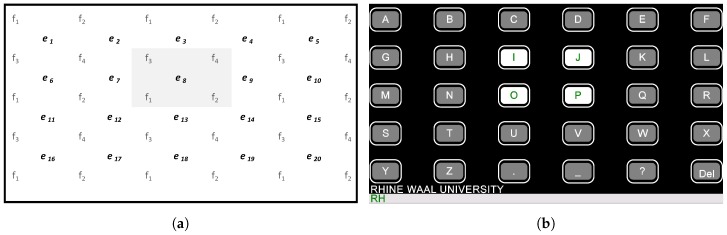
Frequency arrangement for the *Hybrid*. Stimuli $f_{1}=6.00$ Hz, $f_{2}=6.67$ Hz, $f_{3}=7.5$ Hz and $f_{4}=8.57$ Hz were used. Initially one of twenty gaze boxes $e_{i}$ was determined using the eye tracker (**a**). If, e.g., the box $e_{8}$ was classified, the four buttons “I,J,O,P” started to flicker allowing the selection of an individual letter via steady state visual evoked potentials (**b**).

*Hybrid*: The *Hybrid* operated in two phases. Firstly, only eye tracking was utilized, as described above, but instead of a single box, a block of the four nearest boxes to the averaged coordinates were highlighted, as displayed in Figure 3. In total, twenty overlapping blocks were selectable. After three consecutive selections of a block (minimum time 1 s), the corresponding boxes started flickering with four individual frequencies, initiating the second phase. However, gaze coordinates were still calculated in the background. Therefore, if the initial selection was false, the user still could switch to another block of letters by shifting her/his gaze. If the letters of this new block did not overlap with the letters of the preceding block, the flickering stopped. In the occasion that the calculated gaze coordinates were directly in the center of a box, up to four overlapping blocks had the exact same distance to the gaze coordinates. In this case, the lower right block had highest precedence.

To ensure that each block contained four different frequencies, they were arranged as displayed in Figure 3.

The first phase can also be seen as a dynamic gaze shifting period. Flickering started only if the user fixated the block containing the desired letter for a sufficiently long period of time.

In order to increase robustness, for the *Hybrid*, three additional frequencies (means between the four target frequencies) were used as in [34], hence $N_{f}=7$. In particular, the additional frequencies 6.33, 7.09 and 8.13 Hz were considered. However, if one of these frequencies was classified, the output was rejected. This way the reliability of the output was improved, as the risk of false positives e.g., during gaze shifting was considerably reduced. Only if a particular stimulation frequency had the highest probability, exceeded a certain predefined threshold and the classification time window exceeded a certain minimum time period, the corresponding command was classified. As more frequencies needed to be distinguished for the *SSVEP speller*, the minimum classification time windows was set to 20 blocks (approximately 2 s) for this application, in order to avoid false classifications.

**Figure 4:**
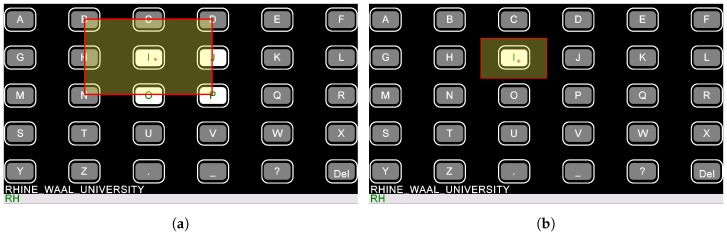
Selecting the letter “I” with the *Hybrid* (**a**) and the *Eyegaze speller* interface (**b**). In the Hybrid system the eye tracking data was used for initial rough selection. If the traced eye coordinates lay within the yellow rectangle (11 cm × 19 cm), the box containing the desired letter started flickering. If eye tracking was used alone, the traced coordinates needed to be much more precise (5.5 cm × 9.5 cm rectangle on the right hand side). The eye tracking software calculated the user’s eye gaze coordinates with an average accuracy of around 0.5° to 1° of visual angle depending on the calibration, which corresponded to an on-screen average error of 0.5 to 1 cm, assuming the user sat approximately 60 cm away from the screen.

EEG data were transferred block-wise to the computer. The minimum SSVEP classification time window for the *Hybrid* was set to 8 blocks (approximately 0.8 s). Figure 4 compares the eye tracking accuracy needed for the *Hybrid* and the *Eyegaze speller*.

### 2.5. Experimental Setup

Initially, participants were prepared for the EEG recording. Thereafter, the eye tracker was calibrated. After accurate positioning in front of the device was ensured, the calibration software provided by the EyeTribe SDK presented a series of calibration targets which were distributed evenly throughout the screen. The calibration process took on average about 30 s to complete. Participants were instructed not to move their head during this calibration phase. Also, participants were asked not to wear their glasses during the experiment, as they affect the performance of the low cost eye tracker system. If the calibration results were poor, re-calibrating was performed.

Afterwards, participants tested the spelling applications as follows: Initially, subjects participated in a familiarization run, spelling the word “KLEVE” and a word of their own choice (e.g., their first name). Next, each participant used each GUI in random order to spell the phrase “RHINE WAAL UNIVERSITY”. The spelling phase ended automatically when the phrase was spelled correctly. In case a person was not able to execute a desired classification within a certain time frame, or if repeated false classifications occurred, the experiment was stopped manually. Spelling errors were corrected via the “delete” button. After the test phase, the subjects completed a post-questionnaire, answering questions regarding each spelling application.

## 3. Results

The overall BCI performance for the three tested spelling applications is provided in Table 2. For each subject, the following values are provided: The time *T* needed to complete the task, the command accuracy *P* and the commonly used information transfer rate (ITR) (see e.g., [1]),

(7) $$B=\log_{2}N+P\log_{2}P+\left(1-P\right)\log_{2}\left[\frac{1-P}{N-1}\right],$$

where *B* represents the number of bits per trial. The overall number of possible choices was N=30 for each application.

The accuracy *P* was calculated based on the number of correct command classifications divided by the total number of classified commands $C_{n}$. To obtain ITR in bits per minute, *B* is multiplied by the number of command classifications per minute. To obtain the average command classification time, the total time needed for the spelling task, *T*, was divided by $C_{n}$.

In some cases, the system was unable to reliably detect the users intent. Participants who were unable to complete the spelling task, or who achieved classification accuracies below 70% were excluded from the calculation of the mean values; these participants, for the sake of brevity, we refer to as BCI illiterates and we define the BCI literacy rate as the percentage of BCI literate participants.

Every participant was able to control at least one of the systems. Out of the 32 participants, 29 were able to gain control over the *Hybrid*, 25 over the *Eyegaze speller* and 24 over the *SSVEP speller*. 18 participants were able to complete the tasks with all three applications. For these subjects a detailed performance comparison was conducted (see Table 3). The typing speed in chars/min was obtained by dividing the total number of spelled letters (including errors and error corrections) by *T*. A series of T-tests revealed that with the *Eyegaze speller* a significant higher ITR than both the *SSVEP speller* (t(17) = 13.924, *p* = 0.000) and the *Hybrid* (t(17) = 11.238, *p* = 0.000) was achieved. On the other hand, the ITR for the proposed *Hybrid* was significantly higher than for the *SSVEP speller* (t(17) = 3.634, *p* = 0.002). Likewise, with the *Eyegaze speller* a significantly higher accuracy compared to the *SSVEP speller* (t(17) = 3.160, *p* = 0.006) and the *Hybrid* (t(17) = 2.747, *p* = 0.014) was achieved. Though the mean accuracy of the *Hybrid* (93.87%) was slightly higher than the *SSVEP speller* (90.81%), the difference was not statistically significant (t(17) = 1.360, *p* = 0.191).

**Figure 5:**
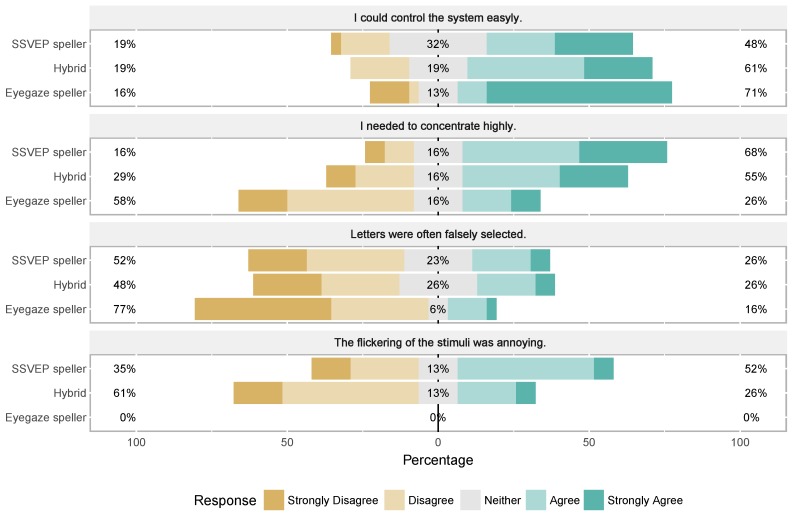
Results from the post test questionaire. Responses were given on a 1-5 Likert scale. The tested applications were the steady-state visual evoked potentials based application (*SSVEP speller*), the here presented *Hybrid* application, and the eye tracking based application (*Eyegaze speller*).

Figure 5 summarizes results from the post questionnaire for all subjects. The subjective impressions regarding user friendliness were measured using a five-point Likert scale, where “1” indicated the strongest degree of disagreement with a particular statement and “5” the strongest degree of agreement.

**Figure 6:**
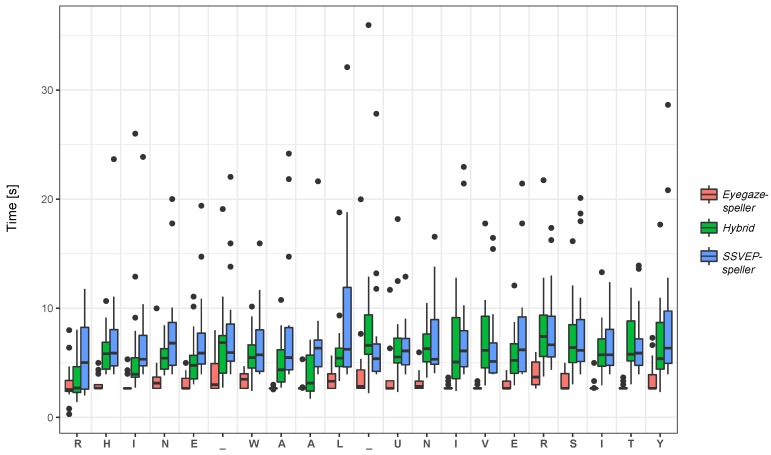
Comparison of letter selection times (only correct selections, i.e., last selection in case of corrected spelling errors). The average classification time needed for every letter of the spelling phrase is provided for each of the tested systems: the steady-state visual evoked potentials based application (*SSVEP speller*), the here presented *Hybrid* application, and the eye tracking based application (*Eyegaze speller*).

Figure 6 compares command classification time for each of the letters form the copy spelling task; the boxplot displays Minimum, Maximum and Median values, as well as Outliers. Note, that in two cases for the *Eyegaze-speller*, classification data was already collected in the background when the recording started and as a result, the first letter was selected in less than one second (e.g., Subject 3 and 5).

**Figure 7:**
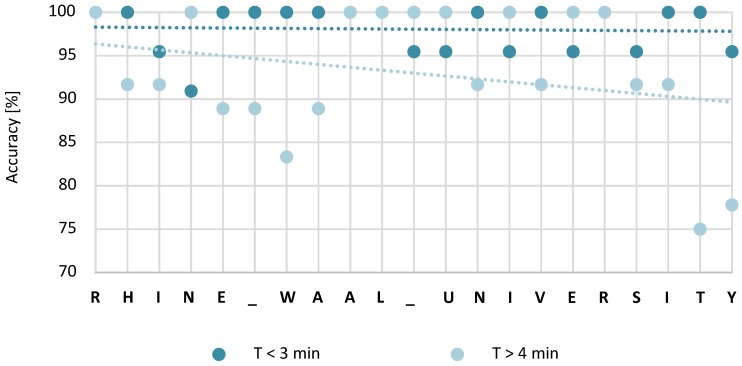
Steady-state visual evoked potentials classification accuracies over the experiment duration for slow and fast performers.

Several participants needed a considerable amount of time to complete the spelling task with the *SSVEP-speller*. In order to analyze the long trial performance, classification accuracies for slow and fast performers were compared. In total, 6 participants needed more than 4 min to write the phrase (slow performers); 11 participants completed the task in less than 3 min (fast performers). Figure 7 suggests that selection accuracy is slightly diminishing over the course of the spelling task for slow performers. To analyze this performance drop further, the classification means of the first and final five letters (94.5% and 87.2%) for slow performers were calculated. The observed difference was not significant (*t*(5) = 1.547, *p* = 0.182)).

## 4. Discussion

The results demonstrate, that while the *Eyegaze speller* was the fastest system overall, the combination of eye tracking and SSVEP showed a faster performance than the SSVEP system alone. All participants gained control over at least one of three systems, yet, the literacy rate differed for each of the systems. In this respect, the proposed *Hybrid* achieved the highest literacy rate; 90.63% of the participants achieved reliable control with the *Hybrid*, 78.13 with the *Eyegaze speller*, and 75% with the *SSVEP speller*.

**Figure 8:**
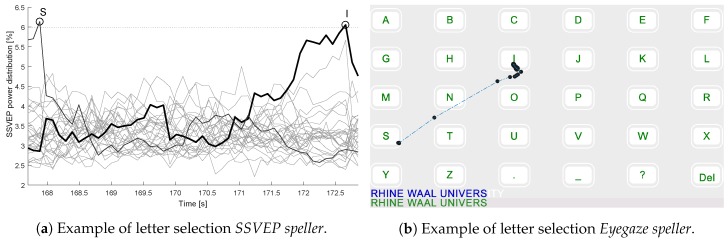
Comparison of the the steady-state visual evoked potentials based application (*SSVEP speller*) and the eye tracking based application (*Eyegaze speller*) during selection of the letter “I” in the example of subject 1. (**a**) The SSVEP power estimations are displayed as a function of time. The black line represents the SSVEP power of the frequency corresponding to the letter “I”. If a certain threshold value (in this case 6) was surpassed, an output command was classified; (**b**) Eye-movement path from the letter “S” to “I”. When the eye focused sufficiently long (1 s) on the desired box, the letter was selected. Before selection, the eye tracker recorded several gaze positions along the path from “S” to “I”.

The speed difference between SSVEP and eye tracking technology was expected. A relatively long time window is necessary until the SSVEP power estimations allow accurate classification. A direct comparison of a letter selection with the *SSVEP speller* and the *Eyegaze speller* is provided in Figure 8.

An explanation why the *Hybrid* was controlled by more users than the *SSVEP speller* is the fewer number of SSVEP targets. This also allowed shorter SSVEP classification time windows and hence resulted in a overall faster performance. For the *Hybrid*, the minimum time for SSVEP classification was below 1 s. On the other hand, for the *SSVEP speller*, we used time windows with minimal length of roughly 2 s, a rather typical value throughout BCI literature (see e.g., [35]). It should be noted though, that some studies successfully used smaller time windows for multi-target systems (see e.g., [31]).

**Figure 9:**
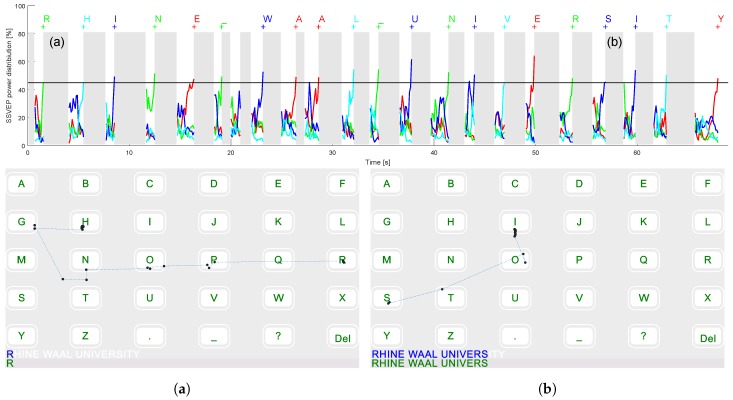
The entire spelling performance for the *Hybrid* in the example of subject 5. The steady-state visual evoked potentials power estimations are displayed as a function of time; the grey boxes represent the eye tracking phases. Eye tracking paths from (**a**) “R” to “H” and (**b**) “S” to “I” are provided.

Another advantage of the *Hybrid* is that the gaze shifting phase was accessed via eye tracking. Therefore, it was ensured that a user is concentrating on a target letter during the collection of EEG data. Figure 9 displays the entire spelling performance of a subject. It can be seen that the gaze shifting period indeed differs for each selection. e.g., for the selection of the consecutive “A”s, the gaze shifting period was as expected the smallest.

A considerable amount of users were not able to control the *Eyegaze speller*. As also observed by Janthanasub and Meesad, the calibration of the eye tracking device was relatively poor or not possible at all for participants with glasses [25]. We like to point out though, that more expensive eye-trackers might perform more reliably when glasses are worn in comparison to the eye-tracker used in our experiment. Future developments in camera based tracking or wearable devices might circumvent this issue. To compare eye tracking and SSVEP independent of the interference of glasses with the tracking, participants were asked to perform this experiment without visual aid, even if usually glasses were worn.

Despite this, 21.87% of the participants were not successful with the eye tracker. Other factors prevented reliable control as well; for example, participant related eye physiology (e.g., narrow eyes) tended to worsen trackability as also observed by Bilignaut and Wium [36]. The Midas touch problem seemed not to be an issue for most participants. Other researchers observed variability among participants in eye tracking performance as well. Räihä and Ovaska also discussed long term use performance [37]. They observed that during a one hour test run with an eye typing system, some participants were unable to complete the experiment due to eye fatigue, while other participants were not affected at all. The authors further listed reasons why eye fatigue may arise: poor calibration, participants frustration, system settings, mental demand, experimental conditions (e.g., temporal demand); also, the use of infrared light over longer time periods may cause discomfort, frustration and dryness of the eye. Eye fatigue can also be an issue with SSVEP-based BCIs (see e.g., [38]). Here, a slightly diminishing accuracy for slow performers was observed (see Figure 7. Apart from fatigue, higher stimulation frequencies towards the end of the phrase could explain the drop.

All in all, subjects gave generally positive feedback regarding the user friendliness of all tested systems (see Figure 5). Regarding the question if the system was easily controlled, the eye tracking system gathered the highest number of extreme answers (strong disagreement/agreement). The perceived level of control for the *Hybrid* was slightly better than for the *SSVEP speller*. Also, the majority of the subjects were more annoyed by the flickering of the *SSVEP speller* compared to the *Hybrid*. Fewer stimuli seemed generally to be less stressful for the user (see e.g., [39]). In addition, the time the subject had to look at a flickering target was larger for the *SSVEP speller*. Higher stimulation frequencies produce less visual fatigue and are more subtle than lower frequencies [40,41] , but their SSVEP amplitudes are significantly lower (see e.g., [27]). Especially for multi-target applications, BCI performance might drop to such an extent that reliable control is not possible. Because of this, we used lower frequencies in the tested applications.

As for the graphical user interface, we decided to use an alphabetically ordered layout as for some users a standard keyboard layout such as QWERTY might be unfamiliar. For people who use a QWERTZ or QWERTY keyboard regularly the interface could be modified. It should be noted, that an equal distribution of selectable targets on the screen might be more efficient in terms of data processing; that is why letters were arranged in a 6 × 5 matrix in the GUI implementation. If e.g., 10 or more letters are used in a row, as typical for the QWERTY keyboard arrangement, the data processing strategy of the hybrid needs to be altered accordingly.

It should be also noted that although the system is designed as a communication tool for disabled people, most of the subjects in this study were healthy young adults. Also, a few of the participants had previous BCI-experience. Therefore, they may not be reflective of the target population. E.g., Käthner et al. stress the importance of engaging end-users during all steps of developement process [42]. They tested eye tracker, electrooculography and an auditory BCI as access methods for augmentative communication. The participant, a 55 year old amyotrophic lateral sclerosis (ALS) patient in locked-in state, rated the ease of use of the auditory BCI as the highest, as no precise eye movements were required, but at the same time as most tiring due to the high level of attention that was necessary to control the BCI. Demographic factors influence BCI performance as well; elderly people for example are slightly poorer BCI performers [43,44]. Future tests with the target population are required.

We also like to mention that while we used a low cost eye tracking device, our SSVEP setup was state-of-art which makes the comparison somewhat biased towards the SSVEP paradigm. However, results from from Kos’myna and Tarpin-Bernard suggest that 4 classes could also be successfully distinguished with a low cost device such as the Emotiv Epoch [17]. A low cost version of the here proposed Hybrid might therefore be possible.

Although slightly slower, the presented SSVEP/eye-tracking combination proofed to be a well-rounded alternative to the pure eye tracking device. The proposed system could however be improved further. In terms of speed, reliability and user comfort, both individual control methods have by far not reached their full potential. But also, their combination offers further improvement possibilities. As recorded EEG signals are affected by non-neuronal activities such as eye blinking and eye movements, eye tracker data can be used to remove such ocular artifacts from EEG signal [45]. Our future work should include this feature as well.

## 5. Conclusions

The article presents a novel eye tracking/SSVEP hybrid spelling application and compares its performance to standalone SSVEP and eye tracking versions of the interface. Generally with eye tracking devices, a large number of targets can be distinguished. With SSVEP-based BCIs high resolution control can also be achieved as demonstrated in several studies. However, due to the high number of distinguishable targets, some users struggle to control either systems. A comparison of mean values revealed that ITR as well as classification accuracy were highest with the pure eye tracking interface, however, the amount of users who gained control was maximal for the proposed hybrid system. It is worth noting, that control over the pure eye tracking interface implied control over the hybrid system, but not vice versa. This indicates that through the data fusion of the two technologies, a wider range of users could access control over hand-free communication applications. Further advantages of the Hybrid system are more dynamic gaze shifting phases between consecutive selections and potentially less expensive hardware in comparison.

## Figures and Tables

**Table 1:** Overview of the tested spelling applications: the setaty-state visual evoked potentials (SSVEP) based application (*SSVEP speller*), the eye tracking based application (*Eyegaze speller*), and the here presented hybrid application (*Hybrid*). The main differences between the three systems are provided.

	No. of Target Characters	No. of Steps for Character Selection	No. of Classes Eye Tracking	Visual Angle Eye Target	No. of Classes SSVEP	Min. Difference between Frequencies	Min. Time Character Selection
*SSVEP speller*	30	1	-	-	30	0.14 Hz	2.031 s
*Eyegaze speller*	30	1	30	$1.6^{\circ }$	-	-	1.000 s
*Hybrid*	30	2	20	$4.4^{\circ }$	4	0.67 Hz	1.813 s

**Table 2:** Results from the copy spelling task of all three tested applications: the steady-state visual evoked potentials based application (*SSVEP speller*), the here presented *Hybrid* application, and the eye tracking based application (*Eyegaze speller*). Participants that were not able to successfully control a spelling interface were excluded from the calculation of mean values for that particular system. The 18 participants who completed the tasks with all three applications are highlighted bold.

Subject	*SSVEP speller*			*Hybrid*			*Eyegaze speller*		
#	Time (s)	Acc. (%)	ITR (bpm)	Time (s)	Acc. (%)	ITR (bpm)	Time (s)	Acc. (%)	ITR (bpm)
**1**	**184.133**	**81.82**	**35.91**	**162.906**	**92.00**	**37.90**	**62.867**	**100.00**	**98.35**
**2**	**188.906**	**78.38**	**36.47**	**112.531**	**88.89**	**55.62**	**64.796**	**100.00**	**95.42**
3	-	-	-	208.508	88.89	30.02	107.111	86.21	59.43
4	-	-	-	321.648	100.00	19.22	-	-	-
**5**	**91.711**	**100.00**	**67.41**	**67.9453**	**100.00**	**91.00**	**56.063**	**100.00**	**110.28**
**6**	**431.641**	**73.33**	**17.36**	**113.953**	**100.00**	**54.26**	**60.125**	**100.00**	**102.83**
**7**	**200.180**	**83.87**	**32.39**	**110.602**	**100.00**	**55.37**	**70.890**	**95.65**	**86.39**
**8**	**114.359**	**100.00**	**54.06**	**117.914**	**100.00**	**52.43**	**61.242**	**100.00**	**100.96**
**9**	**156.914**	**95.65**	**39.03**	**185.758**	**92.00**	**33.24**	**75.563**	**95.65**	**81.05**
10	276.859	86.21	22.99	-	-	-	-	-	-
11	-	-	-	494.406	72.34	15.47	108.010	100.00	57.24
12	-	-	-	677.828	81.82	9.76	64.492	100.00	95.87
13	-	-	-	175.094	86.21	36.35	78.000	92.00	79.16
**14**	**138.531**	**100.00**	**44.63**	**138.734**	**95.65**	**44.14**	**63.477**	**100.00**	**97.40**
15	218.258	100.00	28.33	-	-	-	-	-	-
**16**	**195.609**	**100.00**	**31.61**	**120.352**	**95.65**	**50.88**	**62.359**	**100.00**	**99.15**
**17**	**436.516**	**86.21**	**14.58**	**186.266**	**95.65**	**32.88**	**82.875**	**100.00**	**74.60**
18	92.652	95.65	66.12	108.773	92.00	56.76	-	-	-
19	145.336	95.65	42.14	239.383	82.76	24.76	-	-	-
**20**	**148.891**	**92.00**	**41.47**	**116.289**	**86.21**	**54.73**	**75.766**	**100.00**	**81.60**
21	-	-	-	388.477	76.92	18.11	85.211	92.00	72.46
**22**	**247.406**	**86.21**	**25.73**	**203.633**	**88.88**	**30.74**	**70.891**	**100.00**	**87.21**
**23**	**165.445**	**80.65**	**39.63**	**107.758**	**92.00**	**57.30**	**57.992**	**100.00**	**106.61**
24	1536.760	63.83	4.04	635.375	66.15	14.36	58.703	100.00	105.32
**25**	**231.156**	**85.19**	**25.10**	**239.281**	**88.89**	**26.16**	**71.805**	**100.00**	**86.10**
**26**	**125.227**	**100.00**	**49.37**	**138.531**	**100.00**	**44.63**	**73.836**	**100.00**	**83.74**
27	-	-	-	220.80	95.65	27.74	67.378	100.00	91.76
**28**	**253.297**	**95.65**	**24.18**	**306.008**	**81.82**	**21.61**	**63.172**	**100.00**	**97.87**
29	155.695	100.00	39.71	490.242	74.42	14.97	-	-	-
**30**	**268.125**	**95.65**	**22.84**	**157.219**	**92.00**	**39.27**	**91.914**	**92.00**	**67.17**
31	228.21	100.00	27.09	232.27	86.21	27.40	-	-	-
**32**	**146.047**	**100.00**	**42.33**	**128.375**	**100.00**	**48.16**	**111.475**	**88.89**	**56.15**
Mean	255.115	91.04	34.98	230.229	89.77	37.51	73.840	97.70	86.96
SD	275.114	9.78	14.55	155.121	8.90	17.66	15.622	4.05	15.33
**Literacy** **rate (%)**	**75.00**			**90.63**			**78.13**		

**Table 3:** Mean (SD) values achieved for the 18 participants who completed the tasks with all three applications: the steady-state visual evoked potentials based application (*SSVEP speller*), the here presented *Hybrid* application, and the eye tracking based application (*Eyegaze speller*). The presented values are: the overall accuracy, the information transfer rate (ITR), the characters/minute, an the overall time needed to complete the spelling task). Subjects that were not able to successfully control all three spelling interfaces were excluded from the calculation of mean values.

	Accuracy (%)	ITR (bpm)	Char/Min	Time (s)
*SSVEP speller*	90.81 (8.89)	35.78 (13.36)	8.65 (2.62)	206.894 (96.051)
*Hybrid*	93.87 (5.57)	46.13 (15.75)	10.60 (3.17)	150.781 (56.637)
*Eyegaze speller*	98.46 (3.27)	89.60 (14.14)	18.78 (2.23)	70.950 (13.659)
